# Prescriptions

**Published:** 1898-05

**Authors:** 


PRESCRIPTIONS. *For Erythema-Scratches.*—Menthol, 1 gramme (15 grains); salol, 2 grammes (31 grains); olive oil, 3 grammes (46 grains); lanolin, 80 grammes (122 grains). Apply a small portion to the parts affected three times daily.

*To Counteract Premature Spasmodic Contraction of the Uterus.*—Tinct. iodine, 1 gramme (15 grains); alcohol, 2 grammes (31 grains). Give five drops in a little tepid water every half hour.

*Unguentum Eucaine for Erythema, Inflammation of the Mucous Membrane, and any Painful Swollen Surfaces.*—Eucaine muriate, 1 gramme (15 grains); olive oil, 2 grammes (31 grains); lanolin, 9 grammes. Rub a small portion into the parts three times daily.

*Antiseptic Powder for Dusting on Unhealed Wounds.*—Iodoform, hydrargyri chloridum mite, equal portions of each. Dust a small portion on the affected parts twice daily.

*Unguentum Sozoiodol for Wounds.*—Sozoiodol potassii, 10 parts; lanolin, vaseline, of each, 15 parts. Apply a small portion to the parts affected twice daily.

*For Pruritus of the Skin and Erythema where there is Intense Itching.*—Chloroform, 8 grammes; almond oil, 60 grammes. Apply a small portion to the parts three times daily.—*Recueil de Medicine.*

